# Commentary: Metagenomic next-generation sequencing for *Mycobacterium tuberculosis* complex detection: a meta-analysis

**DOI:** 10.3389/fpubh.2024.1291793

**Published:** 2024-03-15

**Authors:** Xueying Li, Sinian Li, Xuhui Liu, Mutong Fang, Shuihua Lu

**Affiliations:** Department of Pulmonary Disease, Shenzhen Third People's Hospital Affiliated to Southern University of Science and Technology, Shenzhen, China

**Keywords:** metagenomic next-generation sequencing, tuberculosis, mixed infections, infections, molecular diagnosis

We read with interest the meta-analysis by Li et al. (1) on the metagenomic next-generation sequencing (mNGS) for *Mycobacterium tuberculosis complex* detection. mNGS for pathogen detection provides microbial mapping information and can also be used for tailored drug resistance detection, potentially revolutionizing TB diagnosis. Despite this, mNGS is not recommended by many scientific societies or health organizations, including the World Health Organization, as an initial diagnostic tool for TB. Possible reasons for this include the high cost of mNGS and the potential for false-positive results (2).

Based on this analysis, the authors concluded that the specificity of mNGS for TB diagnosis is 100% (95% confidence interval 0.99–1.00). The result is too perfect. From the point of view of the diagnostic technique, we believe that this high specificity is not very reliable and is more likely the result of publication bias. mNGS is also based on the basic principle of bird-needle sequencing, and the primer design is not a particular improvement over other molecular diagnostic techniques. Compared to other validated assays (e.g., Xpert MTB/RIF), mNGS does not technically reduce the possibility of false positives and may introduce more false positives due to contamination of the semi-open assay environment (3, 4). The absence of randomized blinded trials in the cited studies also contributed to the unreliability of the results.

In addition, it is worth note that the analysis showed that the cited studies almost always used composite diagnostic criteria. Some of the studies included mNGS itself in their diagnostic criteria, which is obviously less appropriate for the evaluation of the present study (5); another part did not describe whether mNGS was included or not. We believe that even when analyses are performed using composite diagnostic criteria, comparisons with bacteriologic testing should be provided at the same time.

In addition, it should be noted that the authors mention several times in both the INTRODUCTION section and the CONCLUSION section that mNGS is of great value in areas with a high prevalence of TB, but do not evaluate the strengths and weaknesses of the method compared to other molecular methods. In terms of sensitivity, it is not superior to existing methods (such as the Xpert Ultra) (6) and is much more expensive. The authors cite “a 10% prevalence rate” of the COVID-19 population in Africa, whereas the references are all for the Chinese population, and this misplaced argument is also unreasonable.

mNGS has the property of being a high-throughput assay and may therefore be more suitable for drug resistance detection of *M. tuberculosis* or for differential diagnosis of unexplained infections than for the initial diagnosis of TB. The World Health Organization has also given a positive assessment of the value of targeted NGSfor the detection of drug resistance in *M. tuberculosis* (7), and again does not recommended its use for the initial diagnosis of tuberculosis. Based on the evidence currently available, we believe that further evaluation of the suitability of mNGS for the diagnosis of TB is needed.

## Author contributions

XLi: Writing – original draft, Writing – review & editing. SLi: Investigation, Writing – review & editing. XLiu: Resources, Writing – review & editing. MF: Data curation, Investigation, Writing – review & editing. SLu: Funding acquisition, Resources, Supervision, Writing – review & editing.
